# β-Thalassemia Intermedia: Interaction of α-Globin Gene Triplication With β-thalassemia Heterozygous in Spain

**DOI:** 10.3389/fmed.2022.866396

**Published:** 2022-03-23

**Authors:** Paloma Ropero, Fernando Ataúlfo González Fernández, Jorge M. Nieto, Williana Melissa Torres-Jiménez, Celina Benavente

**Affiliations:** ^1^Hematology Service, Hospital Clínico San Carlos, Madrid, Spain; ^2^Instituto de Investigación Sanitaria Hospital Clínico San Carlos, Madrid, Spain; ^3^Hematology Service, Hospital Virgen de La Luz, Cuenca, Spain

**Keywords:** beta thalassemia intermedia, triplication alpha genes, beta thalassemia, molecular diagnosis, phenotype

## Abstract

**Objectives:**

To verify with hematimetric data that the diagnosis and clinical grade of β-TI can be established when a triplication of alpha genes (α*αα*anti 3.7) and heterozygous β-thalassemia coexist.

**Materials and Methods:**

Retrospective study in which 73 patients of Caucasian origin participated, who simultaneously showed a triplication or quadruplication of genes α and β-thalassemia.

Screening for the most frequent α-thalassemia mutations as well as gene triplication (α*αα*anti 3.7) was carried out by multiplex PCR followed by reverse hybridization with a commercial Alpha-Globin StripAssay kit and confirmed by MLPA (Multiplex ligation-dependent probe amplification). The molecular diagnosis of β-thalassemia was carried out by automatic sequencing according to the Sanger method.

**Results:**

The genotypes have been classified into three groups according to the number of α globin genes and the severity of the alteration in the β globin gene. All had a mutation in the HBB gene (β0-thalassemia, β+-thalassemia severe, and β+-thalassemia mild). Group I patients who have coherent 6 α genes and groups II and III with 5 α globin genes. In group III, the patients were carriers of mutations affecting the β and δ globin genes. The most significant hematological parameters were hemoglobin levels, MCV, RDW, and the percentage of Hb F.

**Conclusions:**

In group I, regardless of the distribution of the 6 α globin genes, homozygous triplication (α*αα*/α*αα*) or heterozygous quadruplication (α*ααα*/αα), the association with heterozygous β-thalassemia results in severe to moderate anemia that may or may not require transfusion therapy, is the severity of the HBB gene mutation that would determine the clinical variation. Group II patients phenotypically behaved like mild thalassemia intermedia, except for one case that presented thalassemic trait because it also presented an associated α-thalassemia (α*αα*/-α3.7). Finally, group III patients behaved as a thalassemic trait since all were carriers of mutations that increase the overexpression of γ genes.

## Introduction

Thalassemic syndromes are the most common genetic diseases worldwide. They comprise a complex, highly heterogeneous group of hemoglobin (Hb) disorders characterized by a defect in the synthesis of one or more globin chains. Depending on which globin chain is decreased or absent, the syndromes are classified as α, β, δ, βδ, γ*δβ*, or ε*γδβ*. They are inherited in an autosomal recessive manner and are characterized by an extreme diversity of phenotypes, making diagnosis a challenge (1, 2).

The spectrum of β-thalassemias is broad, ranging from β-thalassemia major (β-TM), which is characterized by severe anemia from the first years of life to become a severe transfusion-dependent disease, to mild forms that are generally heterozygous with microcytic and hypochromic anemia without obvious clinical manifestations known as β-thalassemia minor, and intermediate forms [β-thalassemia intermediate (β-TI)], which are not transfusion-dependent (3, 4).

The term β-TI was first suggested in 1955 to describe patients with clinical manifestations that were both too severe to be called β-thalassemia minor and too mild to be called β-TM (5).

Although β-TI lacks specific molecular correlates and diagnosis remains largely clinical, a genotype/phenotype association has been described such that genetic modulators determine the genetic basis of phenotypic diversity. Most patients with β-TI are homozygous or compound heterozygous for β-thalassemia, meaning that both β-globin loci are affected and the disease has a recessive genetic pattern. The wide diversity of mutations affecting the β-globin gene (HBB) ranges from mild promoter mutations (mild β^+^-thalassemia) that cause a slight reduction in β-globin chain production, through β^+^-thalassemias, in which β-globin chain production is decreased, to β^0^-thalassemias, which show a complete absence of the β-globin chain. Compound heterozygosity for these mutations provides a wide spectrum of clinical phenotypes (6). Less frequently, involvement of only one β-globin locus is observed, with the other being completely normal; in these cases, β-TI shows autosomal dominant inheritance as in the case of hyperunstable hemoglobins (7). Other genetic modulators are those that are directly involved in the imbalance of the β-globin chains. Homozygosity or double heterozygosity with overexpression of γ-globin chains, either because one or both alleles correspond to a δβ-thalassemia, or because they are associated with molecular alterations in the same or a different one, result in increased γ-globin chain synthesis (8). Also, the increased production of α-globin chains by a triplication or quadruplication of α genes (α*αα*/αα or α*ααα*/αα) associated with a heterozygous β-thalassemia is a β-TI (9) (Table 1).

**Table 1:** Molecular basis of β-thalassemia intermedia.

**Homozygous or double heterozygous β^+^ thalassemic genes**
Combination of a β^0^-thalassemic gene with a mild β^+^-thalassemic gene
Hemoglobins hyperunstable in heterozygous state (dominant β-thalassemia)
Presence of genetic factors that overexpress the γ globin chains ✓ δβ-thalassemia ✓ Hereditary Persistence of Fetal Hemoglobin (HPHF)
Heterozygous β-thalassemia associated with increased production of α-globin chains [(α*αα*/αα), (α*ααα*/αα) or (α*αα*/α*αα*)]
Homozygous or double heterozygous β^0^ or β^+^ thalassemic genes associated with an α-thalassemia

This work aimed to assess, based on hematological parameters and empirical testing, whether the diagnosis and clinical grade of β-TI can be established in cases with both α gene triplication (α*αα*^anti3.7^) and heterozygous β-thalassemia, as α globin gene triplication is an important factor in the severity of β-thalassemia by exacerbating its phenotypic expression and causing an increased imbalance between the globin chains. For this purpose, we present our experience of cases of β-thalassemia associated with α-globin gene triplication (α*αα*^anti3.7^) in Spain over the last 10 years at the Hospital Clínico San Carlos in Madrid.

## Materials and Methods

This retrospective study was conducted from January 2010 to December 2019 and involved 73 patients of Caucasian origin with simultaneous triplication or quadruplication of α-genes and β-thalassemia.

The patients' hematological parameters and reticulocyte counts, including cell morphological analysis, were determined with an automatic cell counter (Coulter LH750 Analyzer; Beckman Coulter, Brea, CA, USA).

HbA_2_ and HbF levels were measured by high-performance liquid chromatography (HPLC) (VARIANT™; Bio-Rad Laboratories, Hercules, CA, USA). Hemoglobin levels were analyzed by zonal capillary electrophoresis (Sebia Capillarys Flex system, Sebia, Norcross, GA) and by ion-exchange HPLC (BioRad Variant II short β-thalassemia program, Bio-Rad, Hercules, CA) following the manufacturers' instructions.

After automated isolation (Biorobot^®^ EZ1; Qiagen GmbH, Hilden, Germany), genomic DNA was quantified with a NanoDrop 1000 (Thermo Scientific, Wilmington, DE, USA).

Screening for the most frequent α-thalassemia mutations as well as gene triplication (α*αα*^anti3.7^) was performed by multiplex polymerase chain reaction (PCR), followed by reverse hybridization with a commercial kit (Alpha-Globin StripAssay, ViennaLab Diagnostic GmbH, Vienna, Austria) and confirmed by multiplex ligation-dependent probe amplification (MLPA) using a commercial kit (SALSA MLPA KIT P140 HBA; MRC Holland, Amsterdam, The Netherlands). Molecular diagnosis of β-thalassemia was performed by automatic Sanger sequencing according to the previously described (10).

In the descriptive study of the data, qualitative variables are presented with their frequency distributions. Quantitative variables are summarized with their means and standard deviation (SD). Quantitative variables with an asymmetric distribution are summarized as medians and interquartile range (IQR). The associations of the parameters between the study groups were assessed using non-parametric Mann–Whitney *U* or Kruskal–Wallis tests for two or more groups, respectively. These non-parametric tests were used because the groups had small sample sizes. For all tests, a significance value of 5% was accepted. Data processing and analysis were performed using IBM SPSS Statistics for Windows, version 2°.

All hematological indices and clinical findings were carried out with the prior informed consent of the patients. In addition, all experiments were conducted in accordance with the principles of the Declaration of Helsinki.

## Results

This study included 73 patients (33 male and 40 female) ranging in age from 5 months to 65 years with heterozygous β-thalassemia associated with α-globin gene triplication (α*αα*^anti3.7^).

Genetically, all patients were carriers of a single alteration in the HBB gene, corresponding to 11 different mutations. These β-gene mutations were: five β^0^-thalassemia (64.4% of patients); two severe β^+^-thalassemia (21.9%) and four mild β^+^-thalassemia (13.7%). The most frequent mutation was the transversion of a C>T in CD 39 of the 2nd exon (β39(C5) Gln>Stop; HBB:c.118C>T), followed by the substitution of a G>A at nucleotide 110 of the first intron (βnt 252 G>A; HBB:c.93-21G>A) (Table 2).

**Table 2:** Mutations identified in the β-globin gene responsible for β-thalassemia.

**Mutation**	**Name HGVS**	**Number of cases (allelic frequency %)**	**Phenotype**
CD39 (C>T)	HBB:c.118C>T	26 (35.6)	β°
IVS-1-nt1 (G>A)	HBB:c.92+1G>A	17 (23.3)	β°
IVS-1-nt110 (G>A)	HBB:c.93-21G>A	15 (20.5)	Severe β^+^
IVS-1-nt6 (T>C)	HBB:c.92+6T>C	4 (5.5)	Mild β^+^
Spanish (δβ)^0^ thal	NG_000007.3:g.60375_153285del92911	4 (5.5)	Mild β^+^
CD82/83 (-G)	HBB:c.251delG	2 (2.7)	β°
CDI (ATG>GTG)	HBB:c.1A>G	1 (1.4)	β°
CD44 (-C)	HBB:c.135delC	1 (1.4)	β°
IVS-II nt-654	HBB:c.316-197C>T	1 (1.4)	Severe β^+^
Hb E	HBB:c.79G>A	1 (1.4)	Mild β^+^
Hb Lepore-Baltimore	NG_000007.3:g.63564_70978del	1 (1.4)	Mild β^+^

The genotypes were classified into three groups according to the number of α-globin genes and the severity of the alteration in the globin-β gene. Group I included four patients with 6 α-globin genes and a mutation in the HBB gene, this mutation was β^0^-thalassemia (two patients), severe β^+^-thalassemia (one patient), and mild β^+^-thalassemia (one patient). Groups II and III included patients with five α-globin genes. Group II comprised 64 patients with β^0^-thalassemia (45 patients), severe β^+^-thalassemia (15 patients), or mild β^+^-thalassemia (4 patients), while group III included patients with mutations affecting the genes β and δ globin and phenotypically categorized as mild β^+^-thalassemia (Table 3).

**Table 3:** β-thalassemia associated with triplications of the α genes.

**Group**	**Patient**	**Age/Sex**	**Hb (g/dL)**	**MCV (fL)**	**MCH (pg)**	**RDW (%)**	**Ret (%)**	**HbA2 (%)**	**HbF (%)**	**N**°**genes α**	**Mutation β gene**	**Severity**	**Phenotype**
I	I	14/M	8.3^*^^§^	57.5	18.7	17.7	4.4	5.0	2.0	α*αα*/α*αα*	CD39 (C>T)	β°	STI
	II	45/M	6.3^*^^§^	88.8	25.9	29.2	2.3	2.8	27	α*αα*/α*αα*	CD82/83 (-G)	β°	STI
	III	33/F	9.8	73.1	23.2	16.3	3.8	3.9	1.0	α*αα*/α*αα*	IVS-I nt-6 (T>C)	Mild β^+^	MTI
	IV	6m/F	9.7	60.8	18.8	22.9	3.1	4.3	3.7	α*ααα*/αα	IVS-I nt-110 (G>A)	Severe β^+^	MTI
II	V	7/F	8.1	60.3	19.8	24.6	3.2	5.2	5.9	α*αα*/αα	CD39 (C>T)	β°	STI
	VI	2/F	8.3	56.2	18.0	19.0	3.0	4.0	5.5	α*αα*/αα	CD39 (C>T)	β°	STI
	VII	30/F	8.3	74.0	22.6	21.2	5.1	4.8	1.2	α*αα*/αα	CD39 (C>T)	β°	STI
	VIII	42/F	8.4	65.6	20.3	17.5	2.3	5.2	3.0	α*αα*/αα	CD39 (C>T)	β°	STI
	IX	5/F	8.5	72.9	22.3	25.8	4.7	3.9	16.0	α*αα*/αα	CD39 (C>T)	β°	STI
	X	16/M	8.6	57.8	18.3	21.6	3.0	5.8	3.0	α*αα*/αα	CD39 (C>T)	β°	STI
	XI	X/M	8.8	60.8	18.8	25.6	2.7	5.5	9.3	α*αα*/αα	CD39 (C>T)	β°	STI
	XII	65/F	8.8	65.9	20.4	23.7	3.0	4.8	1.2	α*αα*/αα	CD39 (C>T)	β°	STI
	XIII	33/F	8.9	62.5	19.8	21.3	2.0	4.3	2.7	α*αα*/αα	CD39 (C>T)	β°	STI
	XIV	32/M	9.1	61.2	19.6	17.1	2.1	4.3	3.5	α*αα*/αα	CD39 (C>T)	β°	MTI
	XV	7/F	9.3	57.8	18.4	18.1	2.4	5.3	1.8	α*αα*/αα	CD39 (C>T)	β°	MTI
	XVI	32/M	9.3	72.0	21.5	21.1	3.0	4.6	10.0	α*αα*/αα	CD39 (C>T)	β°	MTI
	XVII	58/M	9.3	81.3	25.3	26.8	10.4	4.0	10.0	α*αα*/αα	CD39 (C>T)	β°	MTI
	XVIII	23/F	9.7	57.9	18.8	21.5	2.0	4.8	5.7	α*αα*/αα	CD39 (C>T)	β°	MTI
	XIX^**^	29/F	9.8	65.1	19.7	19.2	1.9	5.0	1.6	α*αα*/αα	CD39 (C>T)	β°	MTI
	XX	29/F	9.8	60.6	20.5	11.7	1.2	3.6	1.0	α*αα*/αα	CD39 (C>T)	β°	MTI
	XXI	6/M	9.9	55.8	18.0	21.3	0.8	5.4	7.3	α*αα*/αα	CD39 (C>T)	β°	MTI
	XXII	32/F	10.1	59.3	18.7	16.7	12.0	4.1	2.5	α*αα*/αα	CD39 (C>T)	β°	MTI
	XXIII	59/M	10.4^§^	65.4	20.8	17.3	1.3	4.0	2.8	α*αα*/αα	CD39 (C>T)	β°	MTI
	XXIV	X/M	10.5	63.7	18.2	18.6	1.9	4.9	0.8	α*αα*/αα	CD39 (C>T)	β°	MTI
	XXV	8/M	10.5	65.0	20.0	19.0	0.6	5.6	1.3	α*αα*/αα	CD39 (C>T)	β°	MTI
	XXVI	42/M	10.5	70.4	20.8	23.1	3.7	5.5	3.1	α*αα*/αα	CD39 (C>T)	β°	MTI
	XXVII	3/M	10.6	58.0	19.0	19.8	0.5	5.2	2.1	α*αα*/αα	CD39 (C>T)	β°	MTI
	XXVIII	50/M	10.7	60.2	19.2	17.4	0.8	4.8	0.5	α*αα*/αα	CD39 (C>T)	β°	MTI
	XXIX^***^	X/M	11.1	78.3	23.0	24.9	7.3	5.5	2.7	α*αα*/αα	CD39 (C>T)	β°	MTI
	XXX	20/F	7.2^**^	72.4	20.1	22.5	3.7	4.8	4.0	α*αα*/αα	IVS-I nt-1 (G>A)	β°	MTI
	XXXI	20/F	8.3	73.1	22.6	21.2	5.1	4.8	1.2	α*αα*/αα	IVS-I nt-1 (G>A)	β°	STI
	XXXII	34/F	9.0	64.2	21.2	20.9	2.6	4.2	10.0	α*αα*/αα	IVS-I nt-1 (G>A)	β°	MTI
	XXXIII	60/F	9.1	74.5	26.0	26.0	5.5	5.0	4.5	α*αα*/αα	IVS-I nt-1 (G>A)	β°	MTI
	XXXIV	25/F	9.2	64.4	19.1	16.5	2.1	4.8	3.9	α*αα*/αα	IVS-I nt-1 (G>A)	β°	MTI
	XXXV	6m/F	9.4	61.0	20.0	18.0	1.2	3.6	8.0	α*αα*/αα	IVS-I nt-1 (G>A)	β°	MTI
	XXXVI	23/F	9.6	63.9	21.1	16.5	1.6	5.0	6.0	α*αα*/αα	IVS-I nt-1 (G>A)	β°	MTI
	XXXVII	20/F	9.9	62.1	20.8	14.3	1.1	5.0	3.0	α*αα*/αα	IVS-I nt-1 (G>A)	β°	MTI
	XXXVIII	24/F	10.2	68.5	21.4	19.7	2.6	4.7	10.1	α*αα*/αα	IVS-I nt-1 (G>A)	β°	MTI
	XXXIX	23/M	10.4	60.7	18.4	17.3	1.9	5.0	4.0	α*αα*/αα	IVS-I nt-1 (G>A)	β°	MTI
	XL	24/M	10.4	60.7	18.4	17.3	1.9	5.6	6.0	α*αα*/αα	IVS-I nt-1 (G>A)	β°	MTI
	XLI	18/M	10.4	65.4	20.8	17.3	1.3	4.0	2.8	α*αα*/αα	IVS-I nt-1 (G>A)	β°	MTI
	XLII	X/M	10.5	61.6	19.2	14.8	2.3	4.6	1.5	α*αα*/αα	IVS-I nt-1 (G>A)	β°	MTI
	XLIII	10/F	10.6	57.3	18.7	16.8	0.9	5.0	8.0	α*αα*/αα	IVS-I nt-1 (G>A)	β°	MTI
	XLIV	19/F	10.7	62.7	21.0	16.1	2.2	5.2	2.4	α*αα*/αα	IVS-I nt-1 (G>A)	β°	MTI
	XLV	25/F	11.0	60.0	20.5	15.7	1.1	3.8	1.8	α*αα*/αα	IVS-I nt-1 (G>A)	β°	MTI
	XLVI	26/M	12.5	66.1	21.1	15.4	1.0	5.0	3.0	α*αα*/-α3.7	IVS-I nt-1 (G>A)	β°	TT
	XLIVII	35/F	9.2	66.5	21.7	33.1	4.4	4.0	4.5	α*αα*/αα	CDI (ATG>GTG)	β°	MTI
	XLVIII	X/F	9.0	63.8	18.5	20.9	5.5	3.9	2.0	α*αα*/αα	CD44 (-C)	β°	MTI
	XLIX	12/F	8.8	62.8	19.4	18.4	2.3	5.6	1.1	α*αα*/αα	CD82/83 (-G)	β°	STI
	L	X/F	8.1	63.7	19.7	17.9	1.7	4.8	0.8	α*αα*/αα	IVS-I nt-110 (G>A)	Severe β^+^	STI
	LI	22/F	8.2^**^	63.4	20.6	16.1	2.4	4.3	3.5	α*αα*/αα	IVS-I nt-110 (G>A)	Severe β^+^	STI
	LII	5m/F	8.4	78.7	25.6	18.7	2.5	1.8	21.7	α*αα*/αα	IVS-I nt-110 (G>A)	Severe β^+^	STI
	LIII	21/F	8.6	63.4	20.6	16.1	2.4	4.0	3.0	α*αα*/αα	IVS-I nt-110 (G>A)	Severe β^+^	STI
	LIV	20/F	10.3	61.2	19.6	17.1	2.1	4.2	3.1	α*αα*/αα	IVS-I nt-110 (G>A)	Severe β^+^	MTI
	LV	10/M	10.8	60.3	18.7	16.6	2.0	4.4	1.2	α*αα*/αα	IVS-I nt-110 (G>A)	Severe β^+^	MTI
	LVI	38/M	11.0	59.3	18.5	16.5	3.0	3.7	2.0	α*αα*/αα	IVS-I nt-110 (G>A)	Severe β^+^	MTI
	LVII	24/M	11.6	60.1	17.2	16.2	1.1	5.4	1.8	α*αα*/αα	IVS-I nt-110 (G>A)	Severe β^+^	TT
	LVIII	X/M	12.2	58.7	18.8	16.9	0.9	4.1	0.8	α*αα*/αα	IVS-I nt-110 (G>A)	Severe β^+^	TT
	LIX	27/M	12.4	61.8	19.6	16.6	1.4	5.3	1.4	α*αα*/αα	IVS-I nt-110 (G>A)	Severe β^+^	TT
	LX	X/F	12.9	63.4	20.0	15.5	1.4	4.3	0.7	α*αα*/αα	IVS-I nt-110 (G>A)	Severe β^+^	TT
	LXI	X/M	13.4	62.6	20.2	17.0	1.8	4	1.1	α*αα*/αα	IVS-I nt-110 (G>A)	Severe β^+^	TT
	LXII	X/M	13.5	63.3	20.3	16.7	1.3	4.1	0.4	α*αα*/αα	IVS-I nt-110 (G>A)	Severe β^+^	TT
	LXIII	22/M	13.8	61.0	19.4	15.0	1.5	4.6	1.0	α*αα*/αα	IVS-I nt-110 (G>A)	Severe β^+^	TT
	LXIV	11/M	10.1	60.2	18.4	16.6	2.2	6.6	0.9	α*αα*/αα	IVS-II nt-654 (C>T)	Severe β^+^	MTI
	LXV	X/F	8.3	74.3	20.9	32.7	2.9	3.4	0.5	α*αα*/αα	IVS-I nt-6 (T>C)	Mild β^+^	MTI
	LXVI	X/F	11.3	75.0	22.2	19.4	0.2	3.8	0.3	α*αα*/αα	IVS-I nt-6 (T>C)	Mild β^+^	MTI
	LXVII	60/M	11.4	74.5	23.5	16.2	1.9	3.8	0.9	α*αα*/αα	IVS-I nt-6 (T>C)	Mild β^+^	MTI
	LXVIII	X/M	12.6	68.7	22.2	14.6	0.6	3.8	1.5	α*αα*/αα	Hb E	Mild β^+^	TT
III	LXIX	4/F	9.4	75.8	21.9	24.8	4.4	3.8	14.5	α*αα*/αα	(δβ) Spanish	Mild β^+^	TT
	LXX	49/F	12.1	73.2	23.7	23.0	1.3	2.3	17.2	α*αα*/αα	(δβ) Spanish	Mild β^+^	TT
	LXXI	37/M	12.3	67.7	20.1	23.0	2.2	3.7	8.5	α*αα*/αα	(δβ) Spanish	Mild β^+^	TT
	LXXII	31/M	14.0	71.9	22.2	24.5	1.6	3.0	14.4	α*αα*/αα	(δβ) Spanish	Mild β^+^	TT
	LXXIII	X/F	11.9	66.9	22.2	19.4	2.5	2.5	9.85	α*αα*/αα	Lepore-Baltimore	Mild β^+^	TT

*F, Female; M, Male;*

* *transfusion-dependent;*

** *pregnant;*

§ *deferoxamine chelation;*

*** *splenectomy at 2 years old*.

*STI, Severe Thalassemia Intermedia; MTI, Mild Thalassemia Intermedia; TT, Thalassemic Traits*.

The clinical symptoms of patients who presented severe β-TI (STI) included weakness, conjunctival subictericia, gallstones, splenomegaly, weight loss, and pallor. Patients with mild β-TI mild (MTI) showed slowed growth, delayed puberty and splenomegaly, and bone problems. Patients with thalassemic traits (TT) most often presented fatigue, dizziness, headache, cold extremities, and skin pallor.

From the hematological point of view, β-TI was defined as Hb >7 g/dL. In our study and within each group, the differentiation between STI, MTI, and TT was made according to these levels. Thus, patients with Hb concentrations of 7–9, 9.1–11.5, and >11.5 g/dL were categorized as STI, MTI, and TT, respectively. The distributions according to the groups were as follows: Group I: 2 STI and 2 MTI; Group II: 16 STI, 39 MTI, and 9 TT; and Group III: 5 TT.

The mean values of the hematological parameters of the patients assigned to the different groups and classified as STI, MTI, and TT are shown in Table 4.

**Table 4:** Summary of the hematological data of the different groups according to the phenotype.

	**Group I**		**Group II**				**Group III**
**Phenotype**	**STI** ***N*** **= 2**	**MTI** ***N*** **= 2**	**STI** ***N*** **= 16**	**MTI** ***N*** **= 39**	**TT** ***N*** **= 9**	**Significance** **level**	**TT** ***N*** **= 5**
Hb (g/dL)	7.3 (6.3–8.3)	9.7 (9.7–9.8)	7.8 (7.2–8.9)	10.0 (8.3–11.4)	12.8 (11.6–13.8)	*p =* 0.000^*^	11.9 (9.4–14.0)
MCV (fL)	73.1 (57.5–88.8)	66.9 (60.8–73.1)	65.8 (56.2–78.7)	64.5 (55.8–81.3)	62.8 (58.7–68.7)	*p =* 0.573	71.1 (66.9–75.8)
MCH (pg)	22.3 (18.7–25.9)	21.0 (18.8–23.2)	20.5 (18–25.6)	20.2 (18.0–26.0)	19.9 (17.2–22.2)	*p =* 0.795	22 (20.1–23.7)
RDW (%)	23.4 (17.7–29.2)	19.6 (16.3–22.9)	20.7 (16.1–25.8)	19.2 (11.7–32.7)	16 (14.6–17.0)	*P =* 0.001^*^	22.9 (19.4–24.8)
Retis (%)	3.3 (2.3–4.4)	3.4 (3.1–3.8)	3.1 (1.7–5.1)	2.7 (0.2–12.0)	1.2 (0.6–1.8)	*p =* 0.000^*^	2.4 (1.3–4.4)
HbA2 (%)	3.9 (2.8–5.0)	4 (3.9–4.3)	4.6 (1.8–5.8)	4.5 (3.4–6.6)	4.5 (3.8–5.3)	*p =* 0.811	3.1 (2.3–3.8)
Hb F (%)	14.5 (2.0–27.0)	2.3 (1.0–3.7)	5.2 (0.8–21.7)	3.7 (0.3–10.1)	1.3 (0.4–3.0)	*p =* 0.014^*^	12.9 (8.5–17.2)

*The values of the distribution are expressed by means of the median and interquartile range*.

*Group I: Patients with ααα/ααα and heterozygous β-thalassemia (β^0^ or β^+^)*.

*Grupo II: Patients with ααα/αα and heterozygous β-thalassemia (β^0^ or β^+^)*.

*Grupo III: Patients with ααα/αα ααα/αα and (δβ) Spanish or Hb Lepore-Baltimore*.

*STI, Severe Thalassemia Intermedia; MTI, Mild Thalassemia Intermedia; TT, Thalassemic Traits*.

* *Statistically significant*.

*In group II, the most significant parameters were Hb, Erythrocyte Distribution Width (RDW), the percentage of reticulocytes, and Hb F levels. **Patients with STI** showed hemoglobin levels in a range between 7.2 and 8.9 g/ dL; Mean Corpuscular Volume (MCV) between 56.2 and 78.7 fL; Mean Corpuscular Hemoglobin (MCH) between 18 and 25.6 pg; all presented a high RDW on a scale between 16.1 and 25.8%, and in 9 of them increased reticulocytes. In 15 of the 16 patients, the quantification of HbA2 was elevated with values between 1.8 and 5.8%. HbF increased variably in 12 of them (range 0.8–21.7%)*.

***Patients with MTI** showed, the mean Hb value was 10.0 g/dL (8.3–11.4); the MCV of 64.5 fL with values ranging from 55.8 to 81.3; hypochromia (MCH) between 18 and 26 pg; the RDW was increased in all cases, except one, in a range between 11.7 and 33.1%; in 66% of cases without increased reticulocytes (range 0.2–12%). All presented elevated HbA2, with a mean value of 4.6% (3.4–6.6), and HbF showed a very wide range between 0.3 and 10.1%*.

***Patients classified as TT**, Hb was between 11.6 and 13.8 g/dL, all showed microcytosis (MCV between 58.7 and 68.7 fL) and hypochromia (MCH between 17.2 and 22.2 pg) with reticulocytes. The mean value of HbA2 levels was 4.5% in a range between 3.8 and 5.3% and in HbF, the values ranged between 0.4 and 3%*.

*In group III (5 patients with TT), Hb was 11.9 g/dL between 9.4 and 14 g/dL, microcytosis was established between 66.9 and 75.8 fL and MCH at 22 pg (20.1-23.7 pg); the RDW was greatly increased (22.9%) in a range between 19.4 and 24.8%. Only one presented high reticulocytes (4.4%). HbA2 levels were normal while HbF levels were increased (12.9%). This increase is directly related to the type of patient, since all of them were carriers in a locus of mutations that increase the overexpression of the γ genes, such as the deletion of the δ and β globin genes [(δβ) Spanish] and the gene hybrid δ and β globin (Hb Lepore-Baltimore)*.

## Discussion

The phenotype of thalassemia intermedia involves several factors, including an increased imbalance of α/β globin synthesis as well as the severity of the β-thalassemia mutation.

Our laboratory is a reference center in Spain for the study and molecular diagnosis of structural hemoglobinopathies and thalassemias. We receive ~900 samples annually, all of which are screened for the most frequent α-thalassemia mutations and gene triplication (11). From January 2010 to December 2019, 8,870 samples were received, of which 73 individuals showed both α-gene triplication or quadruplication and β-thalassemia. This is the largest published series of this type of association.

In Spain, the frequency of heterozygous β-thalassemia is ~1.5% while the incidence of α-globin gene triplication is unknown (12). Thus, the association of both entities in Spain is poorly established. Therefore, we collected all cases that showed simultaneous triplication of α-globin genes and β-thalassemia and performed hematological and phenotypical analyses to determine the influence of the increase in alpha genes among patients carrying a single mutation in a β-globin locus on the phenotype of thalassemia intermedia in the Spanish population, compared to previous reports in other countries.

The clinical spectrum of β-TI is very broad, as is the hematologic phenotype. Anemia is the first suspicious sign and can be moderate to mild in patients with β-TI, with adequate red blood cell production to maintain Hb levels >7 g/dL without the need for regular blood transfusions. Transfusions are recommended when Hb drops to <5 g/dL. Some patients are asymptomatic until adulthood or manifest a more benign phenotype. Molecular testing remains the definitive diagnostic tool for thalassemia intermedia phenotype (13, 14).

A total of 11 different mutations among β^0^-thalassaemia, severe β^+^-thalassaemia, and mild β^+^-thalassemia in the HBB gene were identified in our series, with most being β^0^-thalassaemias. The mutations and percentages overlapped with the frequency previously reported in the Spanish population (12).

The four patients in group I had six α-globin genes. Three patients were homozygous for α-globin gene triplication (α*αα*/α*αα*); two had severe anemia, hypersplenism, and osteopathy and required transfusion and chelating treatment with deferoxamine; both also presented β^0^-thalassemia. The third case, without transfusion requirement, was considered MTI, with mild β^+^-thalassemia. The clinical signs included mild anemia, pallor, fatigue, and irritability. The fourth patient also showed a MTI phenotype with a severe β-thalassemia mutation and a distribution of the six α-globin genes corresponding to quadruplication (α*ααα*/αα). The patient was only 6 months old at study inclusion but already showed weight loss, pallor, and splenomegaly and was expected to develop STI with age. Therefore, regardless of the distribution of the six α-globin genes, whether homozygous triplication (α*αα*/α*αα*) or heterozygous quadruplication (α*ααα*/αα), the association with heterozygous β-thalassemia resulted in severe to moderate anemia that might require transfusion therapy, with the severity of the HBB gene mutation determining the clinical variation, as noted by Traeger-Synodinos et al. (15) and Sollano et al. (16). Hematological data are more severe in patients with STI, although no statistical differences were observed in this study due to the small sample size (Table 4).

Group II contained the most patients (64 patients), all of them were carriers of five α-globin genes (α*αα*/αα) except for one, who had a triplication in one allele and had also co-inherited a 3.7 kb deletion (α*αα*/-α^3.7^). This patient, according to the hematological data, was classified as TT presenting with an HBB gene mutation categorized as severe [IVS-1-nt1 (G>A)]; thus, although he has a triplication, when associated with the 3.7 kb deletion, the genetic load of α-globin is 4. Therefore, a patient with a mutation in the HBB gene and four alpha genes would have clinical manifestations and erythrocyte parameters like those for a β-thalassemic trait without complications, as also reported by Villegas et al. in 1997 (17).

In this group, most patients (39/64) showed a MTI phenotype although 70% of the patients were carriers of a β^0^-thalassemia. Thus, we would have expected a higher proportion of patients with a more severe phenotype. Eighteen patients had splenomegaly, 15 had slow growth and delayed puberty, and 8 showed bone problems.

Patients with the STI, MTI, and TT showed significant differences in hemoglobin level, RDW, reticulocyte number, and HbF. The most severe hematological parameters were presented by the 16 patients with STI, with the lowest Hb rate (8.4 g/dL), and the highest HbF (5.2%), RDW (20.7%), and reticulocyte counts (3.1%) (Table 4). These patients also showed hepatosplenomegaly, skeletal lesions, growth retardation, and paravertebral masses.

Subsequent analysis of these statistically significant variables by of 2 to 2 comparisons revealed differences between patients with STI and MTI, STI and TT, and MTI and TT for all variables. For all of these parameters, the values closest to normality were observed in patients with TT, while the most extreme values were observed in patients with STI (Table 5).

**Table 5:** Comparison of means of the significant parameters within group II.

	**Group II**			**Group II**			**Group II**		
**Phenotype**	**STI**	**MTI**	**Significance**	**STI**	**TT**	**Significance**	**MTI**	**TT**	**Significance**
	***N*** **= 16**	***N*** **= 39**	**level**	***N*** **= 16**	***N*** **= 9**	**level**	***N*** **= 39**	***N*** **= 5**	**level**
Hb (g/dL)	7.8 (7.2–8.9)	10.0 (8.3–11.4)	*p =* 0.000^*^	7.8 (7.2–8.9)	12.8 (11.6–13.8)	*p =* 0.000^*^	10.0 (8.3–11.4)	11.9 (9.4–14.0)	*p =* 0.000^*^
RDW (%)	20.7 (16.1–25.8)	19.2 (11.7–32.7)	*P =* 0.056	20.7 (16.1–25.8)	16 (14.6–17.0)	*P =* 0.000^*^	19.2 (11.7–32.7)	22.9 (19.4–24.8)	*P =* 0.003^*^
Ret (%)	3.1 (1.7–5.1)	2.7 (0.2–12.0)	*p =* 0.010^*^	3.1 (1.7–5.1)	1.2 (0.6–1.8)	*p =* 0.000^*^	2.7 (0.2–12.0)	2.4 (1.3–4.4)	*p =* 0.012^*^
Hb F (%)	5.2 (0.8–21.7)	3.7 (0.3–10.1)	*p =* 0.617	5.2 (0.8–21.7)	1.3 (0.4–3.0)	*p =* 0.008^*^	3.7 (0.3–10.1)	12.9 (8.5–17.2)	*p =* 0.007^*^

*STI vs. MTI, STI vs. TT, and MTI vs. TT*.

*The values of the distribution are expressed using the median and interquartile range*.

* *Statistically significant*.

*RDW, Erythrocyte Distribution Width; Ret, Reticulocytes; STI, Severe Thalassemia Intermedia; MTI, Mild Thalassemia Intermedia; TT, Thalassemic Traits*.

Regarding the severity of the β-thalassemia alteration, 73% of cases with a β^0^-thalassemia presented as MTI, with no extramedullary hematopoietic lesions or thromboembolic episodes. Moreover, since periodic transfusions were not required, no hepatic lesions or complications related to ferric accumulation were observed. Half of the patients with severe β^+^-thalassemia (8/15) presented severe or mild TI. Older patients presented leg ulcers, one of the complications of TI, probably due to reduced tissue oxygenation that is believed to occur due to the combination of anemia, hypercoagulability, and inefficient erythropoiesis. Moreover, in mild β^+^-thalassemia, 75% of cases showed an aggravated phenotype (MTI), while 25% presented as uncomplicated TT. These results enrich the data on this type of association previously published in countries of the Mediterranean basin, such as Italy and Greece, as well as in the thalassemic belt, such as India (15, 18–20), where this association is well-established and all patients present as severe or mild β-TI with splenomegaly, delayed growth and hormonal development, and bone problems, and which may require transfusion therapy.

Statistical analysis according to the severity of the β-thalassemia mutation, regardless of phenotype, showed statistically significant differences only for patients with β^0^-thalassemia mutations and severe β^+^-thalassemia, as the number of patients with mild β^+^-thalassemia mutations was very low. The significant parameters were Hb (9.6 vs. 11 g/dL), RDW (19.8 vs. 16.6%), and HbF (4.3 vs. 2.9%), with the most extreme values in patients with β^0^-thalassemia. The hemoglobin level decreased with the severity of the phenotype. The RDW was higher in patients with β^0^-thalassemia because it is a marker for erythrocyte populations are: the more severe the thalassemia, the more marked the anisocytosis (high RDW) with two well-differentiated populations (microcytic and hypochromic erythrocytes and normocytic and normochromic erythrocytes). HbF is increased in β^0^-thalassemia carriers because with less globin-β chain available for αβ dimer formation, excess α-chains will associate not only with δ-globin chains to form HbA_2_ (α2δ2) but also with globin-γ chains for HbF assembly, decreasing the deleterious effect of intracellular precipitation of unbound α-chains. These results confirm that β-TI is more severe when caused by β^0^-thalassemia than when the mutation is due to severe β^+^-thalassemia. Although no significant differences in reticulocytes were observed, despite being increased in all of them, we believe that the excess α-chains contribute not only to HbA_2_ and HbF synthesis but also cause the formation of insoluble tetramers, which precipitate and damage red blood cell membranes. Together with the severity of the β-thalassemia mutation, this condition aggravated the ineffective erythropoiesis, with evident results in the clinic, consistent with the reports by Camaschella et al. and Beris et al. in β-TI (21, 22).

Analysis of differences between STI and MTI based on mutations showed no significant differences except for the CD39 alteration (C>T), which resulted in a lower Hb in patients with STI than in those with MTI, indicating that the hemoglobin level decreases with severity.

The five patients in Group III had both triplication of α-globin genes and a δβ-thalassemia Spanish or a Hb Lepore Baltimore. This type of alterations in the β locus, in which both the δ and globin β genes are compromised, behave phenotypically as a mild β^+^-thalassemia since the increase in HbF synthesis helps to ameliorate the effect of the reduced globin-β chain synthesis. This increase in HbF levels may also show co-adjuvant effects to reduce the phenotypical exacerbation of the triplication of the α-globin genes. All five patients presented microcytosis (71.1 fL), hypochromia (22 pg), normal reticulocyte (2.4%), and HbA_2_ (3.1%) levels, and greatly increased HbF levels (>8.5%), compatible with a TT phenotype including weakness, headaches, and pallor. This type of association was also described by Camaschella et al. in Italy and Altay et al. in Iran. In Italy, the coexistence of heterozygous α-globin gene triplication and a δβ-thalassemia Sicilian or a Hb Lepore Boston presents as β-TI, while in Iran this type of association, as in our series, was described as TT (21, 23).

Statistical analysis of the hematological parameters of patients with TT in group II (mild β^+^thalassemia) and group III (δβ-thalassaemia or Hb Lepore Baltimore) showed differences in all parameters except for HbA and HbA_2_ levels. Both MCV and MCH, as well as RDW, were close to normal levels in group III patients, while reticulocyte and HbF levels were increased (Table 6).

**Table 6:** Comparison of means of hematological parameters in patients with thalassemia trait (TT) of group II and group III.

	**Group II**	**Group III**	
**Phenotype**	**TT** ***N*** **= 9**	**TT** ***N*** **= 5**	**Significance level**
Hb (g/dL)	12.8 (11.6–13.8)	11.9 (9.4–14.0)	*p =* 0.205
MCV (fL)	62.8 (58.7–68.7)	71.1 (66.9–75.8)	*p =* 0.006^*^
MCH (pg)	19.9 (17.2–22.2)	22 (20.1–23.7)	*p =* 0.027
RDW (%)	16 (14.6–17.0)	22.9 (19.4–24.8)	*P =* 0.003^*^
Retis (%)	1.2 (0.6–1.8)	2.4 (1.3–4.4)	*p =* 0.023^*^
HbA2 (%)	4.5 (3.8–5.3)	4.6 (2.3–10.5)	*p =* 0.082
HbF (%)	1.3 (0.4–3.0)	12.9 (8.5–17.2)	*p =* 0.003^*^

*The values of the distribution are expressed using the median and interquartile range*.

* *Statistically significant differences*.

Screening for the most frequent mutations responsible for α-thalassemia and α-globin gene triplication in all patients studied in our laboratory provided the largest series of patients with heterozygous β-thalassemia associated with alpha-globin gene triplication. These data showed that the effect of the association of α-globin gene triplication with a heterozygous β thalassemia mutation is highly variable. The phenotype can range from uncomplicated thalassemic trait to a thalassemia intermedium that can become transfusion dependent. In addition, although the molecular diagnosis of β-TI can be indicative, patients must be evaluated as a whole for an accurate diagnosis of β-TI. For these reasons, molecular diagnosis and genetic counseling are important since, although the heritability of a triplication of alpha genes is in many cases underdiagnosed and does not present clinical complications, it can be co-inherited with another pathology, such as heterozygous β-thalassemia, which can aggravate the condition. Transfusion therapy may be required owing to all complications that this type of therapy entails, as well as the clinical sequelae resulting from ineffective erythropoiesis and anemia observed in intermediate thalassemia, although today there are new therapies such as luspatercept (erythroid maturation agent) to combat anemia. In our country, it is administered for compassionate use, since it is not yet approved by the government and in the sporadic cases that have been treated, good results have been obtained, with very good efficacy and safety and with no adverse effects.

## Data Availability Statement

The original contributions presented in the study are included in the article/supplementary material, further inquiries can be directed to the corresponding author/s.

## Ethics Statement

The studies involving human participants were reviewed and approved by Comite Etica Asistencia Sanitaria GAE Clinico San Carlos. Written informed consent to participate in this study was provided by the participants or their legal guardian/next of kin.

## Author Contributions

All authors listed have made a substantial, direct, and intellectual contribution to the conception and design of work, drafting and revising for intellectual content, and final approval of the version to be published. All authors contributed to the article and approved the submitted version.

## Conflict of Interest

The authors declare that the research was conducted in the absence of any commercial or financial relationships that could be construed as a potential conflict of interest.

## Publisher's Note

All claims expressed in this article are solely those of the authors and do not necessarily represent those of their affiliated organizations, or those of the publisher, the editors and the reviewers. Any product that may be evaluated in this article, or claim that may be made by its manufacturer, is not guaranteed or endorsed by the publisher.

## Abbreviations

β-TM: β-thalassemia major
β-TI or TI: β-thalassemia intermediate
Hb: Hemoglobin
HBB: β-globin gene
HPLC: High-performance liquid chromatography
HPHF: Hereditary Persistence of Fetal Hemoglobin
IQR: Interquartile range
MCH: Mean Corpuscular Hemoglobin
MCV: Mean Corpuscular Volume
MLPA: Multiplex ligation-dependent probe amplification
MTI: Mild β-TI mild
RDW: Erythrocyte Distribution Width
Ret: reticulocytes
SD: Standard deviation
STI: Severe β-TI
TT: Thalassemic traits.

## References

[1] Higgs DR Engel JD Stamatoyannopoulos G. Thalassaemia. Lancet. (2012) 379:373–83. 10.1016/S0140-6736(11)60283-3 21908035

[2] Musallam KM Rivella S Vichinsky E Rachmilewitz EA. Non-transfusion-dependent thalassemias. Haematologica. (2013) 98:833–44. 10.3324/haematol.2012.066845 23729725 PMC3669437

[3] Galanello R Origa R. Beta-thalassemia. Orphanet J Rare Dis. (2010) 5:11. 10.1186/1750-1172-5-11 20492708 PMC2893117

[4] Vitrano A Calvaruso G Lai E Colletta G Quota A Gerardi C. The era of comparable life expectancy between thalassaemia major and intermedia: is it time to revisit the major-intermedia dichotomy? Br J Haematol. (2017) 176:124–30. 10.1111/bjh.14381 27748513

[5] Sturgeon P Itano HA Bergren WR. Genetic and biochemical studies of intermediate types of Cooley's anaemia. Br J Haematol. (1955) 1:264–77. 10.1111/j.1365-2141.1955.tb05509.x 13240015

[6] Galanello R Cao A. Relationship between genotype and phenotype. thalassemia intermedia. Ann NY Acad Sci. (1998) 850:325–33. 10.1111/j.1749-6632.1998.tb10489.x 9668554

[7] Weatherall DJ Clegg JB. The Thalassemia Syndromes. Oxford: Blackwell Science (2001) 827 p. 10.1002/9780470696705

[8] Sankaran VG Lettre G Orkin SH Hirschhorn JN. Modifier genes in Mendelian disorders: the example of hemoglobin disorders. Ann NY Acad Sci. (2010) 1214:47–56. 10.1111/j.1749-6632.2010.05821.x 21039591

[9] Steinberg MH Forget BG Higgs DR Weatherall DJ. Disorders of Hemoglobin: Genetics, Pathophysiology, and Clinical Management. New York, NY: Cambridge University Press (2009) 826 p. 10.1017/CBO9780511596582

[10] Torrejón MJ Ortíz-Cabrera NV M Nieto J González Fernández FA Villegas A Martínez R. Hb Moncloa: a new variant of haemoglobin that interferes in the quantification of Hb A1c. Clin Biochem. (2017) 50:521–4. 10.1016/j.clinbiochem.2017.04.014 28433609

[11] de la Fuente-Gonzalo F Nieto JM Villegas A González FA Martínez R Ropero P. Characterization of deletional and non-deletional alpha globin variants in a large cohort from Spain between 2009 and 2014. Ann Hematol. (2019) 98:1537–45. 10.1007/s00277-019-03696-w 31025160

[12] Villegas A Ropero P González FA Anguita E Espinós D. The thalassemia syndromes: molecular characterization in the Spanish population. Hemoglobin. (2001) 25:273–83. 10.1081/HEM-100105220 11570720

[13] Capellini MD Cohen A Porter J Taher A Viprakasit V (editors). Guidelines for the Management of Transfusion Dependent Thalassaemia, 3rd Ed. Nicosia: Thalassemia International Federation (2014). 25610943

[14] Origa R Baldan A Marsella M Borgna-Pignatti C. A complicated disease: what can be done to manage thalassemia major more effectively?. Exp Rev Hematol. (2015) 8:851–62. 10.1586/17474086.2015.1101339 26470003

[15] Traeger-Synodinos J Kanavakis E Vrettou C Maragoudaki E Michael T Metaxotou-Mavromati A. The triplicated alpha-globin gene locus in beta-thalassaemia heterozygotes: clinical, haematological, biosynthetic and molecular studies. Br J Haematol. (1996) 95:467–71. 10.1046/j.1365-2141.1996.d01-1939.x 8943886

[16] Sollaino MC Paglietti ME Perseu L Giagu N Loi D Galanello R. Association of α globin gene quadruplication and heterozygous β thalassemia in patients with thalassemia intermedia. Haematologica. (2009) 94:1445–48. 10.3324/haematol.2009.005728 19794088 PMC2754962

[17] Villegas A Muñoz JA Risueño CF Castro JM Sánchez J Ropero P. Association of alpha and beta thalassemia with alpha gene triplication in one family. Med Clin. (1997) 108:781–3. 9265084

[18] Camaschella C Mazza U Roetto A Gottardi E Parziale A Travi M. Genetic interactions in thalassemia intermedia: analysis of β-mutations, α-genotype, γ-promoters, and β-LCR hypersensitive sites 2 and 4 in Italian patients. Am J Hematol. (1995) 48:82–7. 10.1002/ajh.2830480203 7847345

[19] Theodoridou S Balassopoulou A Boutou E Delaki EE Yfanti E Vyzantiadis TA. Coinheritance of triplicated alpha-globin gene and beta-thalassemia mutations in adulthood: ten years of referrals in northern Greece. J Pediatr Hematol Oncol. (2020) 42:e762–4. 10.1097/MPH.0000000000001730 32032239

[20] Mehta PR Upadhye DS Sawant PM Gorivale MS Nadkarni AH Chandrakala S. Diverse phenotypes and transfusion requirements due to interaction of β-thalassemias with triplicated α-globin genes. Ann Hematol. (2015) 94:1953–8. 10.1007/s00277-015-2479-8 26319530

[21] Camaschella C Kattamis AC Petroni D Roetto A Sivera P Sbaiz L. Different hematological phenotypes caused by the interaction of triplicated alpha-globin genes and heterozygous beta-thalassemia. Am J Hematol. (1997) 55:83–8. 10.1002/(SICI)1096-8652(199706)55:2&<83::AID-AJH6&>3.0.CO;2-Z 9209003

[22] Beris P Solenthaler M Deutsch S Darbellay R Tobler A Bochaton-Pialat ML. Severe inclusion body beta-thalassaemia with haemolysis in a patient double heterozygous for β^0^-thalassaemia and quadruplicated alpha-globin gene arrangement of the anti-4.2 type. Br J Haematol. (1999) 105:1074–80. 10.1046/j.1365-2141.1999.01451.x 10554822

[23] Altay C Öner C Öner R Gümrük F Mergen H Gürgey A. Effect of α-gene numbers on the expression of β-thalassemia intermedia,β -thalassemia and (δβ)^0^-thalassemia traits. Hum Hered. (1998) 48:121–5. 10.1159/000022792 9618059

